# A Survey on Node Clustering in Cognitive Radio Wireless Sensor Networks

**DOI:** 10.3390/s16091465

**Published:** 2016-09-10

**Authors:** Gyanendra Prasad Joshi, Sung Won Kim

**Affiliations:** Department of Information and Communication Engineering, Yeungnam University, 280 Daehak-Ro, Gyeongsan, Geongbuk 38541, Korea; joshi@ynu.ac.kr

**Keywords:** node clustering, cognitive radio, cognitive radio wireless sensor networks

## Abstract

Cognitive radio wireless sensor networks (CR-WSNs) have attracted a great deal of attention recently due to the emerging spectrum scarcity issue. This work attempts to provide a detailed analysis of the role of node clustering in CR-WSNs. We outline the objectives, requirements, and advantages of node clustering in CR-WSNs. We describe how a CR-WSN with node clustering differs from conventional wireless sensor networks, and we discuss its characteristics, architecture, and topologies. We survey the existing clustering algorithms and compare their objectives and features. We suggest how clustering issues and challenges can be handled.

## 1. Introduction

Communications in wireless sensor networks (WSNs) is event-driven (i.e., whenever an event is triggered, wireless sensor (WS) nodes generate bursty traffic). Conventionally, WSNs are designed to work on a single channel with non-rechargeable/irreplaceable batteries and a very low active duty cycle. In a dense network environment, WS nodes deployed in the same area might try to access a channel whenever a new event triggers communications, which results in scarce bandwidth to send data packets from all the nodes in the region. These days, an increasing number of sensitive and critical activities are being monitored and observed using bandwidth-hungry multimedia WSNs. Cognitive techniques are now being used in wireless sensors to circumvent the bandwidth limitations imposed by conventional WSNs. Integration of cognitive devices in WS nodes extends their capability to utilize unused spectrum and provides greater bandwidth. The cognitive radio wireless sensor network (CR-WSN) is a candidate for the next generation of WSN systems [1,2,3,4].

Wireless sensors are normally deployed in inaccessible terrain. Therefore, the self-configuring, self-organizing abilities and the lifetimes of the WS nodes have become very important. Along with these capabilities in sensor nodes, the following are the basic characteristics of cognitive radio wireless sensor (CR-WS) nodes.

- Nodes can work with multiple channels.
- Nodes listen and follow instructions from the base station.
- They can switch channels within a pre-specified time.
- They broadcast/unicast spectrum information to neighbors.
- They feature spectrum sensing, analyzing, predicting, decision making, and management.

Details about CR-WSNs were outlined by Joshi et al. [1]. In this paper, we use the terms spectrum and channel, and node clustering and clustering interchangeably.

Most of the WSNs that measure physical phenomena, like temperature, humidity, location and movement of objects, etc., are delay-tolerant and require low bandwidth. Therefore, sensors with CR capabilities are basically not required. CR-WSNs are generally required for monitoring systems where low delay, high throughput, and reliability are essential. These requirements are especially applied to multimedia applications.

Logically grouping and consolidating similar sensor nodes in their proximity with certain objectives is called node clustering. A clustered wireless sensor networking architecture is advantageous to a non-cluster-based architecture in various ways. A non-cluster-based architecture is also called a single-tier network architecture and is based on a flat topology (discussed in Section 3). Node clustering enables bandwidth reuse and efficient resource allocation; thus, it can improve system capacity. Particularly, in a large scale and a dense sensor network, a single-tier network can overload the gateway node, leading to congestion and delay in communications. The single-tier network is not scalable for a larger set of sensors deployed over a large area.

Clustering in CR-WSNs is still in its infancy. There has been plenty of work done in clustering for mobile ad hoc networks (MANET) [5,6], WSNs [7], and cognitive radio networks (CRNs) [8,9]. Although some clustering issues have been addressed in the literature [10,11,12,13,14,15,16,17,18,19,20,21,22,23,24,25,26,27,28], clustering still remains a vastly unexplored domain in CR-WSNs.

This work attempts to explore the requirements of node clustering in CR-WSNs, compares node clustering in CR-WSNs and WSNs, and discusses the issues and challenges of node clustering in CR-WSNs. The goals of this work are as follows: (1) to make a large audience aware of the requirements of clustering schemes in CR-WSNs; (2) to provide information about the differences and similarities between clustering in WSNs and CR-WSNs; (3) to encourage researchers to propose suitable clustering algorithms for CR-WSNs, because this is a new research area, and is still in its infancy; (4) to highlight a few strengths and weaknesses of the existing algorithms; and (5) to provide a comprehensive analysis of the clustering mechanism and encourage proposals for the best strategy.

In this paper, the challenges and essence of clustering in CR-WSNs are described and discussed. Clustering in conventional WSNs is described in Section 2. Characteristics, architectures, and topologies are explained in Section 3. Objectives and advantages of node clustering in CR-WSNs are discussed in Section 4. Comparison of clustering in CR-WSNs and WSNs is presented in Section 5. Section 6 surveys the existing literature. Issues and challenges are detailed in Section 7. Finally, Section 8 concludes the paper.

## 2. Clustering in Conventional WSNs

Because wireless sensor nodes are battery-operated tiny devices, energy conservation has significant importance when designing the physical layer for WSNs. They are deployed in harsh environmental conditions, and the sensor devices are inaccessible after deployment. Therefore, it is not always possible to replace the sensors’ batteries. Valid, accurate, and reliable data collection basically depends on the sensors’ lifetime, which is determined by residual energy of the system. Hence, the most important challenge in WSNs is the efficient use of energy resources.

In WSNs, energy consumed for sensing and computation is much lower than for communications [29]. Therefore, a majority of the total energy consumed in WSNs is transmission power, and the required transmission power grows exponentially with an increase in transmission distance. In general, the minimum output power required to transmit a signal over distance *d* is proportional to *d^n^*, where *2* ≤ *n* < *4*. The exponent *n* is closer to 4 for low-lying antennae and near-ground channels, as is typical in wireless sensor network communications [30]. Therefore, routes that have more hops with shorter hop distances can be more power-efficient than those with fewer hops but longer hop distances. Clustering encourages sensor nodes to communicate in multihop fashion, which extends the lifetime of WS networks. If the node density is high enough, it can also effectively overcome shadowing and path loss effects.

There are several strategies in the literature to conserve energy and extend WSN lifetime, such as data reduction [31], protocol overhead reduction [7], reducing the duty cycle [32], energy-efficient routing design [33], energy-efficient MAC design [10,34], and energy harvesting [35,36]. These strategies reduce energy consumption by controlling and mitigating the sensing process, the communications process, collisions, overhearing, control packets, idle listening, and interference [7,31,32,33,34]. Among these strategies, the ability to harvest energy from the environment is highly dependent on many environmental factors. The current state of technology in energy harvesting is still unable to provide a sustained energy supply to enable WSNs to operate continuously [35,36].

Clustering has the advantages of load balancing and scalability, and can extend WSN lifetime when the network size grows. In clustering, wireless sensor nodes are usually grouped into physically disjointed, and often non-overlapped, clusters. All the contiguous nodes are in one or more clusters according to a cluster formation algorithm. Based on the clustering schemes, there is a leader in each cluster, often called a cluster head (CH). The CH is responsible for data aggregation, information dissemination, and network management, and the cluster members (CMs), i.e. non-cluster head nodes, are responsible for event sensing and information collection from their surroundings.

**Figure 1:**
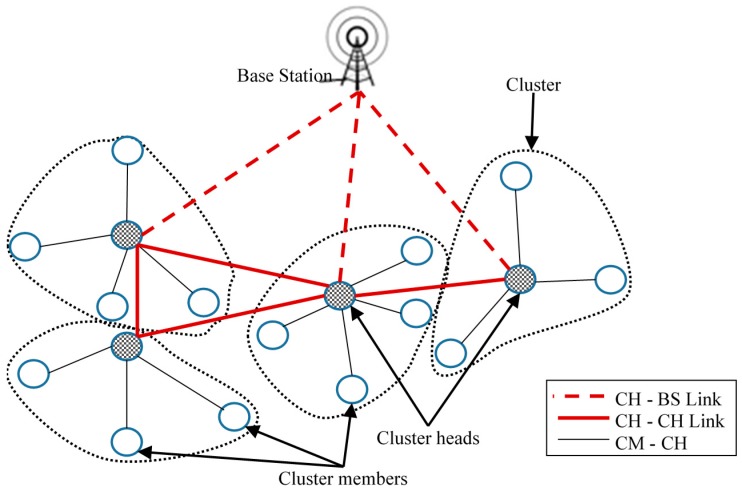
Conventional typical WSN cluster structure.

CHs perform the task of intra- and inter-cluster management and data processing. Some of the responsibilities of CHs include coordination between inter-clusters and intra-clusters, such as cluster formation, data aggregation, and communications with the base station (BS). Each WSN consists of one or more sinks or base stations collecting information from sensor nodes. Figure 1 shows a conventional clustering structure of a typical WSN. As shown in Figure 1, sensor nodes are divided into a number of virtual groups (dotted lines) based on predefined clustering schemes. Inside a cluster, a sensor node may have a different status or function, such as a CH, or a CM. Several types of clustering exist, such as static, dynamic, single-hop and multihop, homogeneous and heterogeneous.

## 3. CR-WSN Characteristics and Architecture

### 3.1. Characteristics of CR-WSNs

The existing clustering algorithms in the literature [11,12,13,14,15,16,17,18,19,20,21,22,23,24,25,26,27,28] consider homogeneous CR-WS nodes, and CHs are selected iteratively based on residual energy and other parameters. Most of these clustering methods are similar to conventional clustering for WSNs, and seldom consider the complexities of CR-WSNs.

In CR-WSNs, the CH should continuously monitor the channel status, i.e. whether the channel is occupied by any incumbent users, also called primary users (PUs). The CH receives spectrum information from the BS and neighbor nodes in its vicinity, and negotiates and allocates available channels. Therefore, the CH requires dedicated hardware, and conventional methods of CH selection that are applied in WSNs (based on geographic region, load, residual energy, links, etc.) do not provide enough parameters. Nevertheless, the methods applied by the conventional CH selection algorithms could be considered when choosing an ideal place to deploy a cluster head (where it can cover the maximum number of neighbor sensor nodes) or when determining the shortest distance from the base station, among other things.

Resources in WSNs are greatly constrained in terms of power supply, processing capability, and transmission bandwidth. Different from WSNs, a CH in a CR-WSN may need to be powered by a heavy-duty battery. Additionally, processing capability, memory, etc., should be enhanced significantly.

It is better to design a global addressing scheme, such as Internet Protocol (IP) for better protection of the license users’ right to access the incumbent channels. With a global addressing scheme, it is easy for the BS to know which wireless sensor node is using which particular channel in certain geographical location. This information helps BS to allocate a channel that has less probability of PUs’ arrival, which mitigates the collision probability with the PUs and protects the right of PUs to access the channel. Applying IP in WSNs is difficult, because address updating in a large-scale or dynamic WSN can result in heavy overhead. However, CR-WSNs can be designed with IP addresses, because the CH has special hardware. Table 1 summarizes the differences between clustering in WSNs and CR-WSNs.

### 3.2. Types of CR-WSNs

Based on the types of nodes deployed, CR-WSNs can be categorized into two kinds.

#### 3.2.1. All WS Nodes Equipped with CR (CR-WSN-all)

In this type of CR-WSN, all WS nodes are equipped with a CR module. These CR-WS nodes, also called secondary users (SUs), in the network sense available spectrum within a particular region and report to the base station (BS) or an intermediate node. The existing clustering algorithms in the literature consider this type of CR-WSN. Node deployment and clustering is easy in this type of network. However, it is costlier in terms of form factor and energy consumption.

#### 3.2.2. Few Nodes with CR Ability (CR-WSN-few)

In this type of network, only few nodes have a CR module, and other nodes have only limited CR or no CR module. If the performance of a CR-WSN-all and a CR-WSN-few is similar, embedding a CR module in each sensor with higher hardware and energy costs is unnecessary. CR-WSNs in which only CHs are equipped with CR for local spectrum sensing and channel assignments could be a better approach.

In CR-WSN-few, nodes with a CR module work as a coordinator node (a cluster head after cluster formation). They sense the local spectrum and report to the BS. That node broadcasts the channel selection decision to other neighbor nodes. This type of network is advantageous in terms of cost and energy consumption. However, coordinator node deployment is difficult, and may create a single point of failure.

### 3.3. Node Deployment

Deployment of CR-WS nodes directly affects the network-setup cost and spectrum information-gathering capability of the CR-WSN. Node deployment is a critical task, as it should be optimal in order to increase network lifetime, PU protection, and cost-effectiveness. The deployment cost depends upon the number of WS nodes with CR that are deployed in the particular area. Basically, CR-WS nodes can be deployed in two ways: deterministic and non-deterministic.

#### 3.3.1. Deterministic Deployment

This is also called structured deployment, or regular deployment. This type of deployment is done in an indoor environment or in a small and favorable environment. All types of CR-WSNs (see Section 3.2) can be considered for this type of deployment, which provides the best possible coverage, and allows easier clustering. Common types of deterministic deployment strategies are the triangular grid, the rectangular grid, and the hexagonal grid. The coverage area of each node in triangular grids, rectangular grids, and hexagonal grids are $\left(\sqrt{3}/4\right)R^{2}$, $R^{2}$, and $\left(3\sqrt{3}/4\right)R^{2}$, respectively, where R is the distance between any adjacent nodes. The total number of nodes required to cover a given area, A, in a triangular grid is $\left\lceil \frac{4A}{\sqrt{3}R^{2}}\right\rceil$, in a rectangular grid, is $\left\lceil \frac{A}{R^{2}}\right\rceil$, and in a hexagonal grid, is $\left\lceil \frac{4A}{3\sqrt{3}R^{2}}\right\rceil$. Deterministic deployment is recommended for a fixed location where fewer sensors are deployed. It is easy to maintain, and has lower maintenance costs. For uninterrupted performance, multiple CHs should be deployed.

#### 3.3.2. Nondeterministic Deployment

This is also called unstructured deployment or random deployment. Generally, sensor networks are targeted at inaccessible, unattended, and possibly hostile environments. Therefore, sensor nodes are deployed by relatively uncontrolled means, such as being dropped by a helicopter, or released from an airplane. Examples of such deployment models are uniform distribution and Poisson distribution. Assuming that *N* WS nodes are uniformly distributed over area *A*, the WS node density can be given by λ = *N/A*. In a Poisson distribution, the probability that there are *m* WS nodes within area *S* is $P(m)=\frac{{\left(\lambda S\right)}^{m}}{m!}e^{-\lambda S}$ [37]. This type of deployment is recommended for non-reachable places, such as deep forests, within the sea, in harsh deserts, on mountains, or in remote areas. A CR-WSN-all (see Section 3.2) is recommended for this type of network, because deployment of a CH that covers all the nodes in the network is difficult to achieve.

With the CR-WSN-few type, multiple CH-capable nodes should be deployed for the CH and backup CH nodes.

### 3.4. Classification of CR-WSNs According to Network Topology

The two basic types of topology used for dissemination and gathering are the flat topology and the hierarchical topology.

#### 3.4.1. Flat Topology

In this type of network, the BS directly aggregates data using routing algorithms. The BS broadcasts channel availability information to its members using a common control channel (CCC), and the CR-WS nodes hop to the channel and communicate with the BS via multihop routes. Based on the types of node deployed, the following two types of flat topologies can be considered for CR-WSNs.

(a) Flat CR-WSN with All CR-WS Nodes (Flat CR-WSN-all)

This is similar to traditional WSNs, where the BS usually broadcasts a query message, and the WS nodes that have data matching the query will send a response to the BS. The BS updates spectrum availability information (SAI) from the spectrum database for the particular geographic region that is maintained by the authority providing the local or central spectrum information. The BS finds the common channels between the SAI and its own sensing result, and then broadcasts a channel availability message to the CR-WS nodes in its vicinity. The CR-WS nodes again compare the channel list received from the BS to their own channel-sensing results and select the best channel to communicate with the BS.

**Figure 2:**
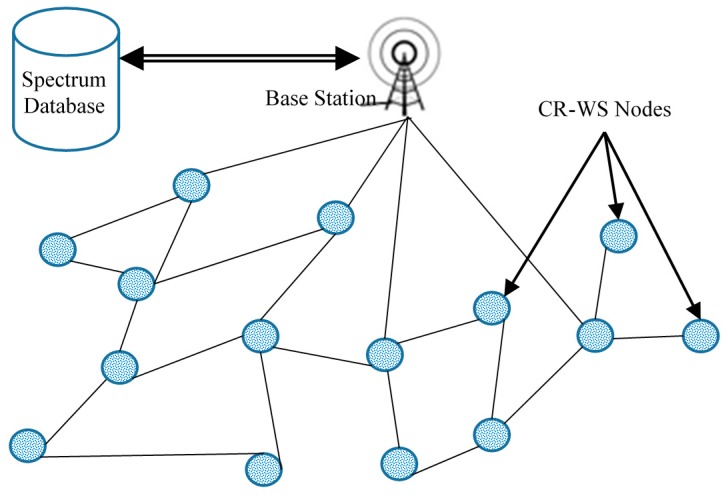
Flat network topology with all CR-WS nodes.

Figure 2 shows the flat network topology. The WS nodes usually communicate in multihop fashion by using peer nodes as relays. This network is very expensive in terms of deployment cost and energy efficiency. This type of network has a scalability issue, and suffers from the energy hole problem. Therefore, it is recommended for a small geographic region.

(b) Flat CR-WSN with Only Few CR-WS Nodes (Flat CR-WSN-few)

This is similar to a Flat CR-WSN-all network; however, only few nodes have CR module, unlike the previous category. All nodes do not participate in the channel-sensing and channel-selection processes. Only the nodes with CR module sense channels and send available channel information to the BS, and all other nodes just follow the BS’s broadcast message for channel selection and channel switching. In this network, “node with CR module” deployment with proportional representation for all targeted geographical reason is difficult. However, overall hardware cost is comparatively inexpensive, because not all WS nodes require CR modules.

#### 3.4.2. Hierarchical Topology

Here, data aggregation is accomplished by structuring CR-WS nodes into clusters. A node that is responsible for data aggregation and fusion is called a cluster head. Data aggregation and fusion at the CH reduces the number of messages transmitted to the BS, and hence, mitigates energy consumption and conserves bandwidth by reducing the number of nodes taking part in transmissions.

Conventionally in WSNs, clustering is based on several parameters, such as node deployment, cluster count, inter-cluster communications, node and CH mobility, node types, node roles, node identifiers, node connectivity, neighborhood information, the cluster formation algorithm, the power conservation method, CH roles, and the CH selection method. The classification of clustering methods in WSNs has been well studied [32,38,39,40,41,42,43,44].

In addition to the above-mentioned parameters, clustering in CR-WSNs should consider the types of nodes, as described in Section 3.2. Based on the types of nodes deployed, there are two types of hierarchical CR-WSN.

(a) Hierarchical CR-WSN with All CR-WS Nodes (Hierarchical CR-WSN-all)

**Figure 3:**
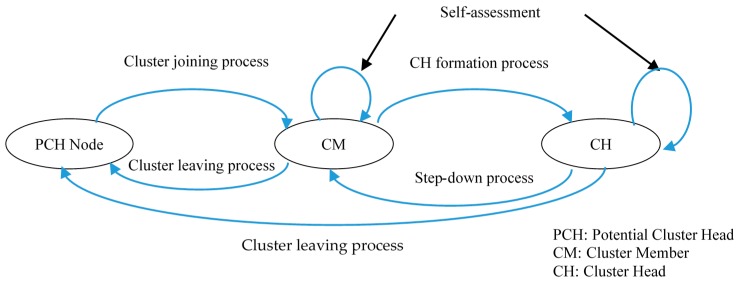
States of CR-WS potential cluster head (PCH) nodes.

This is similar to traditional hierarchical WSNs, where sensor nodes are organized into clusters, and the CHs serve as relays for transmitting the data. CHs may work for data fusion and reduce data transmission redundancy. CHs are selected from potential cluster head (PCH) nodes. Figure 3 illustrates the states of CR-WS PCH nodes.

Although clustering algorithms and CH-selection algorithms are similar to traditional WSNs, CR-WSNs consider one or more cognitive parameters, such as the number of idle channels available to a node, and the maximum number of nodes connected to a channel. Although deployment is easy in this topology, it is expensive in terms of energy efficiency and hardware costs.

(b) Hierarchical CR-WSN with Only CHs as CR-WS Nodes (Hierarchical CR-WSN-few)

In this type of CR-WSN, WS nodes are organized into clusters, and only CHs are equipped with CR. Other WS nodes go without CR. CHs have dedicated hardware with different transmission ranges, and are generally stationary.

CHs are responsible for receiving instructions from the BS and the surrounding CHs about the available spectrum, and they allocate channels for inter-cluster and intra-cluster communications. CHs gather spectrum information by sensing, and they make a channel-selection decision by selecting the best available channel for communications with CMs and the BS. CMs select and hop to the channel ordered by the CHs in order to switch on and start communications. Because the CHs have a higher transmission capacity than CM nodes, the minimum requirement for the number of clusters can be derived from the lower bound of the throughput.

This type of CR-WSN is more reasonable in hardware costs. Because only CHs have CR and serve without changing roles, they require better battery backup. Energy harvesting by the CHs, and additional CHs for backup in every cluster, can help prolong network lifetime. In this type of network, CH deployment is difficult.

To the best of our knowledge, there is no study on CR-WS node-clustering algorithms that considers hierarchical CR-WSN-few along with other cognitive parameters, such as connectivity, coverage, number of CMs, spectrum availability, and, very importantly, PU protection.

**Figure 4:**
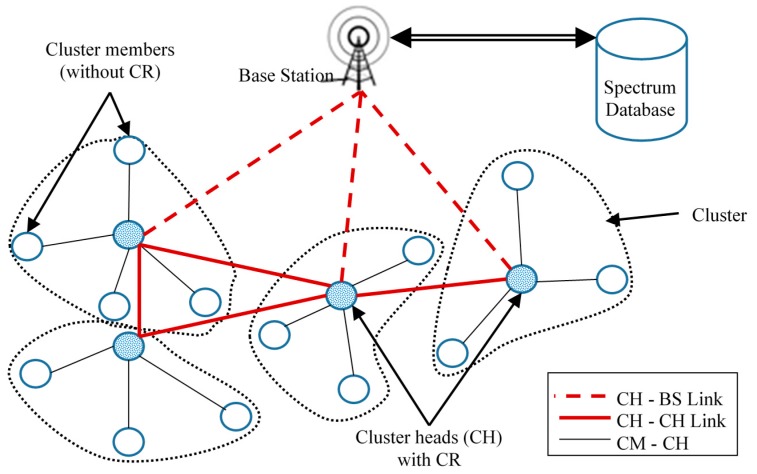
Hierarchical network topology for a CR-WSN where only the CHs are CR-WS nodes.

A typical hierarchical network topology for a CR-WSN where only CHs are CR-WS nodes is shown in Figure 4.

Regardless of the topology used, the BS receives spectrum information from the spectrum database, its own sensing, and the CR-WS nodes.

## 4. Objectives and Advantages of Node Clustering in CR-WSNs

Clustering in CR-WSNs distributes network-wide spectrum sensing and allocation issues to small groups, which solves their issues in the local area. Cluster members contribute to spectrum sensing that helps collect spectrum information from that particular area [1,19]. More information about spectrum availability reduces interference with PUs of the channels.

Clustering in CR-WSNs pursues the following objectives: (a) better local spectrum information; (b) efficient resource allocation; (c) mitigation of interference with incumbent license holders, the PUs, and cognitive users (or secondary users); (d) protect the PUs’ right to access the spectrum; (e) reduce the cost of equipping all sensors with CR; (f) provide network scalability; (g) implement energy conservation; (h) reduce communications overhead; and (i) obtain better data aggregation.

The following are the advantages of clustering in large-scale and dense CR-WSNs.

### 4.1. Scalability

Scalability is the ability of a CR-WSN to adapt to its own expansion while improving efficiency. The CR-WSN is said to be scalable if it accommodates more CR-WS nodes at a later stage, after the design. Clustering in CR-WSNs efficiently deals with problems within a local cluster, and resolves issues over resource limitations. It is easy to keep localized (distributed) spectrum sensing records and update spectrum information, which is the key success factor for communications in CR-WSNs. Clustering also helps to localize the route set up within the particular geolocation, which reduces the size of the routing table stored in the individual sensor nodes. Compared with a flat topology, this kind of network topology is easier to manage, and is more scalable when responding to events in the environment. An important condition for scalability is to keep the average distance between source and destination nodes small enough as the network size grows.

### 4.2. Increased Connectivity

Connectivity is the presence of a single- or multiple-hop path between any source and destination nodes in the CR-WSN. Network connectivity determines the efficiency of multihop routing used in CR-WSNs. Connectivity affects the network capacity, and it depends on various factors, such as interference, signal-to-noise ratio (SNR), and energy constraints [42,43]. To increase network efficiency, these issues should be solved in a local effort within the cluster.

### 4.3. Licensed User Protection

The availability of electromagnetic spectrum has a geospatial correlation. The incumbent spectrum available in one geographic location may not be available in another geographic location. Hence, it is necessary for CR-WS nodes to share spectrum information among neighbors.

Grouped CR-WS nodes deployed in the same geographic region may collect and share more information about the spectrum resource available in that particular region, which helps reduce the chances of PUs and SUs contending for the same channel at the same time. Therefore, clustering helps mitigate interference with the PUs, and protects the rights of PUs.

### 4.4. Energy Conservation

Although energy consumed for sensing and computation in WSNs is much lower than for communications, this is not the case in CR-WSNs. In CR-WSNs, CR-WS nodes have to sense and monitor channels, maintain the available-channel information, predict channel status for future use, relay the sensing report to the CH or BS and its neighbors, and listen to the signal of the CH, BS, and neighbor nodes for spectrum information. Therefore, CR-WS nodes have a higher duty cycle. CR-WS nodes may have multiple transceivers to work on multiple channels, especially one for a control channel and another for data channels. These transceivers must switch channels frequently; hence, they consume more energy.

Because a frequent role change for a CH is not always possible in CR-WSNs, as described in Section 3, preserving the energy of the CHs as well as the CMs, is necessary.

In clustering, inter-clustering communications is performed by the cluster head. Therefore, the number of sensor nodes performing long distance communications decreases. Furthermore, as described in Section 3, clustering encourages multihop communications, which conserves energy. Therefore, clustering is an effective way to conserve energy in CR-WSNs.

### 4.5. Spectrum Reuse and Bandwidth Utilization

Multihop clustering is appropriate for large-scale CR-WSNs. In large-scale CR-WSNs, resources can be allocated orthogonally to each cluster for collision mitigation. The resources can be reused, cluster by cluster. Because clustering limits the scope of inter-cluster interactions to CHs, and circumvents message exchange redundancy among CR-WS nodes, it can also conserve communications bandwidth.

CHs with a CR capability deployed in small clusters may collect better information about local spectrum availability. This provides opportunities to cognitive users (also known as SUs) to utilize the bandwidth, which helps achieve the CR-WSN goal to efficiently utilize spectrum unused by PUs.

### 4.6. Time Synchronization

Basically, spectrum information is gathered by cooperative sensing in CR-WSNs. In cooperative sensing, all CR-WS nodes in the network share the same quiet period, and sense the spectrum simultaneously. Time synchronization is indispensable, because a coordinated and simultaneous quiet period is used for spectrum sensing and time frame/slot allocation.

Actually, reliable signal detection is only possible by fusing sensing results from multiple sensors in the presence of shadow fading [44]. Therefore, all the clocks in a CR-WSN should be synchronized. Time differences can cause nodes to sense the spectrum at different times; this may lead to incorrect sensing results, and the presence of primary users may be overlooked.

Furthermore, time synchronization enables the use of time division multiple access (TDMA) techniques that are generally considered more efficient than contention-based media access control (MAC) layer techniques. Many MAC schemes for CR-WSNs may need time frame/slot structures, and require precise time synchronization for efficient operation. Some applications may also require strict time synchronization, such as tracking. Clustering can provide significant support for perfect synchronization by overcoming any inherent out-of-synchronization deficiency.

### 4.7. Reducing Network Traffic by Data Aggregation and Fusion

Data aggregation and fusion are the processes of aggregating the data from multiple sensor nodes and providing fused data to the BS. Data aggregation and fusion methods eliminate redundant transmissions, hence saving bandwidth and energy.

In densely deployed CR-WSNs, many sensors produce very similar data. Transferring similar data by all CR-WS nodes may generate network-wide massive traffic that wastes network bandwidth and other resources. In clustering, CR-WS nodes send data to the CH that keeps localized traffic within the cluster. Each CH aggregates data, and transmits the fused data to the BS or another CH. Generally, CHs form a tree structure to transmit aggregated data via multihops through other CHs, which results in significant energy savings [45].

### 4.8. Fault-Tolerance and Robustness

CR-WSNs are prone to failure for various reasons, such as energy depletion, hardware malfunctions, and malicious attacks. Clustering helps to apply a fault-tolerant approach locally, without affecting the complete network. Hence, the network becomes more robust, and easy to maintain and manage. Clustering provides more convenience for network topology control, and responds to channel availability changes, PU arrival, and unpredicted node failures.

### 4.9. Reduced Overhead and Delay

After clustering, not every node needs to forward data to the BS, which helps to mitigate network traffic overload, data packet collisions, and retransmissions. It also reduces frequent channel switching, and hence, reduces delay. Frequent channel switching in a CR network was described by Joshi et al. [46]. *C*lustering stabilizes the network topology at the sensor level, and reduces topology maintenance overhead.

Besides the applications of clustering mentioned above, clustering can be employed to perform attacker detection [47]. Because CR-WSN utilizes idle spectrum resources, it is vulnerable to selfish attack. In the attack, one or more selfish CR-WS nodes occupy spectrum that they are not using [48,49,50].

## 5. Node Clustering in WSNs vs. CR-WSNs

Although the objective of clustering in CR-WSNs is similar to that in WSNs (i.e., scalability, coverage, extended lifetime, robustness, better data collection, and aggregation), clustering in CR-WSNs is more challenging, because PUs are unlikely to cooperate with SUs. New cognitive radio network-related challenges are node deployment, CH selection, PU arrival prediction, channel allocation, increased complexity, error rate, and PU interference.

WSNs are generally designed for a single channel, whereas CR-WSNs require multiple channels. If any PU claims the channel currently used by CR-WS nodes, the channel must be vacated immediately within a prespecified time.

In addition to the clustering challenges faced by WSNs, CR-WSNs have to protect the right of the incumbent users to access licensed channels. The responsibilities of the CHs and CMs are different in CR-WSNs than in WSNs. It is even more difficult to handle unpredictable and frequent channel-status changes in CR-WSNs. Therefore, clustering schemes for WSNs cannot be directly implemented in CR-WSNs.

Trust and security should be more controlled in CR-WSNs, because some malicious or selfish nodes can intrude on the communications by interfering with the licensed channels or not changing the channel within the prespecified time period. In addition, a malicious node might report false sensing results about the channel status.

Workload for a CH in CR-WSNs is much greater than in WSNs. CHs in CR-WSNs have to focus on inter-cluster and intra-cluster communications, as well as take care of available channel information in the central spectrum database and channel availability information from neighbors and/or any other CHs. This workload is unnecessary in WSNs.

In WSNs, a basic parameter for CH selection is residual energy. In case of energy depletion, the CH responsibility switches to another node, so that a CH’s energy depletion does not break network connectivity. On the other hand, in CR-WSNs, if CHs have dedicated hardware, network lifetime depends upon the CH’s energy. Therefore, energy conservation for the CH is important. The active duty cycle of nodes in WSNs is very low (as low as 1%) [51]. However, the active duty cycle is high in CR-WSNs, because CR-WS nodes have to monitor PU activities and report to the CH.

## 6. Survey of Some of the Existing Node Clustering Techniques

Many node clustering schemes have been proposed in the literature for the different objectives and goals.

Xu et al. [10] proposed a cluster-based MAC protocol called KoN-MAC for CR-WSNs. It is based on a clustering scheme for WSNs by Lin and Gerla [52]. Pritom et al. [11] proposed a MAC protocol for cluster-based CR-WSNs, but its MAC scheme is based on the Low-Energy Adaptive Clustering Hierarchy (LEACH) protocol [53], which is specially designed for WSNs.

A novel clustering-based spectrum sensing (CBSS) in CR-WSNs was proposed by Qu et al. [12]. In this method, sensor nodes are grouped into different sets based on their similarity in sensing results. An objective function is proposed to identify the optimal cluster number. Details for cluster formation and CH selection were not given, and the performance was not compared with existent algorithms.

Rauniyar and Shin [13] proposed a clustering scheme for cooperative spectrum sensing based on other approaches [20,23]. In the scheme, a pair of nodes in a group can alternate between sleep and wake modes during the sensing process.

A spectrum-aware cluster-based energy-efficient multimedia (SCEEM) routing protocol was proposed by Shah et al. [14]. In this protocol, SU nodes form a cluster with a higher number of commonly available idle channels. The CHs are selected based on energy and relative spectrum awareness, such that noncontiguous available spectrum bands are clustered and scheduled to provide continuous transmission opportunities.

Salim et al. [15] proposed clustering with temporary support nodes (CENTRE). This scheme has a fixed cluster formation duration. It assumes that CMs adjust transmission power based on distance to the CH.

Mustapha et al. [16] proposed an energy-efficient spectrum-aware reinforcement learning-based clustering (EESA-RLC) algorithm for CR-WSNs. In this algorithm, a member node learns the energy and cooperative sensing costs for neighboring clusters in order to achieve an optimal solution.

Manoor and Shahid [17] proposed a clustering scheme called BECHR for CR-WSNs. This scheme selects a CH based on residual energy in an iterative manner. It works like conventional WSN clustering methods but with some variations.

Eletreby et al. proposed a spectrum aware clustering protocol for CR-WSNs called CogLEACH [18]. It is an extension of the LEACH protocol, and uses the number of vacant channels as a weight in the probability for each node to become a CH. This work evaluates CogLEACH in three different models. It extends the throughput and lifetime of the network, compared to the regular LEACH protocol that operates in the same settings.

Zhang et al. [19] and Han et al. [21] proposed distributed spectrum-aware clustering (DSAC) for CR-WSNs based on group-wise constrained (GWC) agglomerative clustering (GCAC) [54]. The basic idea is to set each node as a disjoint cluster at the beginning, and to merge the two nearest clusters during each iteration until the cluster number is reduced to the optimal number. During each iteration, the inter-cluster distances are recalculated. Initially, DSAC considers each node as a CH, and then merges CHs within each iteration until the number of CHs reaches the optimum number.

Mustapha et al. [20] proposed an energy-aware clustering (EAC) algorithm.

Ozger et al. [23] and Ozger and Akan [55] proposed event-driven spectrum-aware clustering (ESAC) and mobility-aware Event-to-sink Spectrum-Aware Clustering (mESAC) protocols that form temporal clusters for each event in CR-WSNs. These protocols determine eligible nodes for clustering based on the local position of nodes between the event and the sink. CHs are selected from among the candidate nodes based on node degree, channel availability, and distance to the sink. Because this is event-driven clustering, the clusters are immediately dismissed after finishing data transmission. Hence, it is not suitable for other scenarios.

Pei et al. [24] proposed adaptive clustering for CR-WSNs called low-energy adaptive uneven clustering hierarchy (LEAUCH) for cognitive radio sensor networks. LEAUCH elects candidate CH nodes based on the number of idle channels, and selects a CH based on distance from the sink. To avoid the energy hole among CHs, it employs an uneven-sized clustering method (i.e., fewer members in the clusters near the sink).

Zubair et al. [25] proposed a distributed solution to establish a common control channel in a geographic area using spatial spectral correlation for virtual clustering for CR-WSNs. It reduces route failure and energy consumption.

Nguyen and Koo [26] studied clustering for CR-WSNs. This work formulates the throughput maximization problem as mixed-integer nonlinear programming to find optimal sensor clustering and to utilize the Branch and Bound algorithm.

Shah and Akan [27] proposed a spectrum-aware cluster-based routing (SCR) protocol for CR-WSNs. Nodes are clustered based on their relative spectrum awareness and residual energy. The protocol selects CHs based on energy and relative spectrum awareness, such that non-contiguous available spectrum bands are clustered and scheduled to provide continuous transmission opportunities.

Park et al. [28] proposed a cognitive radio-based hybrid data-type clustering (CR-HDC) algorithm for CR-WSNs. It uses blocking and forced termination probabilities for CH transmission range and channel occupancy.

General objectives of clustering in CR-WSNs should include CCC establishment, stability, energy efficiency, cooperative tasks, and PU protection. Clustering metrics should be channel availability, geographic location, signal strength or channel quality, number of optimal clusters or cluster heads, etc. Many existing clustering schemes are extensions of clustering schemes for WSNs, with some performance enhancements, such as a lower number of clusters, lower clustering overhead, lower energy consumption, etc.

Table 2 summarizes the existing clustering schemes in the literature regarding their objectives, the metrics they use for clustering, performance enhancements, and CH selection methods.

The clustering algorithms described in Table 2 consider all nodes as being equipped with CR. Many of them have no CCC or rendezvous notion. These protocols do not consider PU protection as one of the main objectives. CHs do not have dedicated hardware, and are mostly iteratively selected based on residual energy and number of idle channels.

## 7. Issues and Challenges of Node Clustering in CR-WSNs

Along with the challenges faced by WSNs, CR-WSNs have to adapt to frequent environmental changes, such as the available bandwidth, the interference level, fading, path loss, etc. Adaptation to environmental changes includes adaptive channel coding, source coding, and modulation. It also requires using multiple channels for seamless communications. Therefore, they face more challenges than WSNs. Particularly, single point failure, proportional deployment of nodes with CR module, and backup node selection are new challenges to be addressed in CR-WSN-few. The fundamental design considerations of CR-WSNs should be as follows.

### 7.1. Broadcast Support

CR-WSNs should support broadcasting, because WS nodes need to listen for emergency signals, and leave the channel whenever incumbent license holders claim it. Additionally, some applications, such as routing protocols, use broadcast information. Many WSNs use passive sensors with an omnidirectional antenna, where all nodes communicate with each other over the same channel. Therefore, it is easy for them to support broadcast information. However, different nodes may communicate on different channels in CR-WSNs, and they may not receive broadcast information. This may cause higher delay and/or network partition.

### 7.2. Busy Receiver Problem

The busy receiver problem may occur in CR-WSNs because of the multichannel network. Transmitters cannot find their receivers on a channel because the receivers are busy on other channels (either transmitting or receiving). Thus, the busy receiver problem increases the dropped packet rate and wastes channel bandwidth.

### 7.3. Control Channel Saturation Problem

In CR-WSNs, one dedicated channel (or one dedicated control time duration) is necessary to communicate with other sensors. A problem occurs in a dense network environment when the network load increases. The control channel becomes the bottleneck preventing the data channels from being utilized efficiently. This problem is also called the control channel bottleneck problem [58].

### 7.4. Multichannel Hidden Node Problem

CR-WSNs have to cope with both the single-channel hidden node problem and the multichannel hidden node problem. To mitigate the traditional and well-known single-channel hidden node problem, request-to-send (RTS) and clear-to-send (CTS) handshakes are used. However, if nodes utilize multiple channels, a node may miss channel negotiation packets, because they are busy communicating on another channel, and the multichannel hidden node problem occurs. Details of the multichannel hidden node problem can be found elsewhere [59,60].

### 7.5. Fault-Tolerance

In order to avoid the loss of significant data from nodes, fault-tolerant CHs are usually required. Re-clustering is considered the most intuitive method to recover from a cluster failure. However, re-clustering is difficult in CR-WSNs, especially if only the CH has dedicated hardware. Deploying multiple CHs for backup is a viable solution for CH failure.

### 7.6. Energy Hole Avoidance

In a large-scale CR-WSN, CR-WS nodes forward packets in multihop fashion to deliver the collected data to a BS. The nodes closer to the BS have to receive and retransmit more packets generated by the sensors than those farther away from the BS. This leads to energy depletion in the nodes closer to the BS, leaving a hole near the BS called an energy hole. This phenomenon partitions the whole network, and prevents outside nodes from sending information to the BS, even though many nodes in CR-WSNs still have plenty of energy. It is difficult to solve the energy hole problem completely; however, it can be mitigated by efficient node deployment, energy harvesting, and efficient CH selection strategies.

### 7.7. Single Point of Failure

With CR-WSN-few, the CH has special hardware and is relatively more vulnerable to CH failure (also called the single point of failure) than in a CR-WSN-all.

### 7.8. Node Deployment

Clustering in CR-WSNs brings several deployment challenges, such as ensuring PU access rights, SU connectivity, selection of the optimal channel, CH selection (under CR-WSN-all), CH deployment (under CR-WSN-few), and optimal cluster sizes. For measuring an accurate degree of optimum coverage, sensing PU activities, and maintaining connectivity, an optimal strategy for node deployment is essential. Node deployment could be different based on the objectives, such as: (1) PU protection; (2) coverage and spectrum information collection; (3) minimal energy consumption configuration; (4) reduction of event-sensing delay; and (5) reduction of the control channel saturation problem.

### 7.9. Optimal Number of Clusters

One of the challenges in cluster-based CR-WSNs is to determine the optimal number of clusters. A large number of clusters leads to establishing long routes, which naturally incur higher delay. On the other hand, a small number of clusters exhausts the energy of the CH, and incurs inefficient spectrum sharing.

The cluster establishment procedure and its complexity should be independent of the network scale, and should remain constant. A fixed cluster size has load-balancing and energy-consumption issues. In particular, CHs that are closer to the BS need to forward more inter-cluster packets, and hence, will deplete their battery power sooner than the CHs that are farther away from the BS. Forming relatively small clusters near the BS distributes the workload among multiple CHs.

When CHs are specialized CR-WS nodes (under CR-WSN-few), there should be a CR-WSN with fewer CHs, because the CHs tend to be more expensive and bigger in size than the CMs. These nodes can be easily visible and detectable if deployed for critical application fields, such as battlefields, border security, infrastructure security, etc.

### 7.10. Connectivity

Connectivity is one major goal of node clustering in CR-WSNs. Ideally, a CM is able to communicate with its CH, either in one hop or in multiple hops, in intra-cluster communications. In inter-cluster communications, nodes can communicate via gateway nodes or via CHs.

### 7.11. Node Synchronization

Synchronization ensures CR-WS node activities start simultaneously in the whole network. Therefore, in distributed clustering algorithms, node synchronization is very important for better performance. Lack of synchronization may result in a suboptimal choice of data channels and may interfere with PU activities. Although some synchronization-based clustering methods for CR-WSNs exist in the literature [14,18], getting perfect synchronization is difficult.

Detailed challenges of CR-WSNs are well studied in [1].

## 8. Conclusions and Future Direction

In large-scale CR-WSNs, clustering is an effective way to achieve goals like obtaining information on local spectrum, efficient resource allocation, interference mitigation, PU protection, coverage and deployment cost reduction, network scalability, communications overhead reduction, an extended lifetime, robustness, and better data collection and aggregation. Clustering is very important in CR-WSNs because it provides a topology control approach to reduce transmission overhead and to exploit data aggregation among a large number of CR-WS nodes. Clustering in CR-WSNs is different from conventional clustering algorithms in WSNs in various ways. CR-WS nodes in CR-WSNs are responsible for channel sensing, channel allocation, data collection and data aggregation, including other cognitive tasks. Cluster heads in clustering help data aggregation and fusion, which intensely reduces the number of nodes taking part in packet transmission, and prolongs the overall network lifetime.

In a small indoor or a favorable area, where the BS is very close to the sensors (typically, just one hop), a flat CR-WSN can be advantageous. That is because the BS is at least two hops away after clustering. Clustering also increases cluster formation and maintenance overhead.

One critical step in clustering is to deploy the CHs uniformly to conserve energy, and mitigate interference with the PU of the channel. If cost, size, and energy are not the issue, most existing clustering algorithms for WSNs, which are primarily focused on scalability and energy conservation, can be tailored to CR-WSNs. The primary focus of the algorithm should be protection of the PU’s right to access the channel. Otherwise, designing CR-WSNs with only the CH having a CR module can be the best way.

Clustering in CR-WSNs brings several unique challenges to deployment, such as mitigating interference with PUs, ensuring connectivity among WS nodes, determining optimal cluster sizes, etc. Clustering in CR-WSNs is still in its infancy, and several key issues are yet to be addressed, such as interference mitigation, the optimal number of CHs and backup CHs, cooperative sensing with and without clustering, among others. Although the optimum number of clusters in WSNs has been addressed by many researchers, to the best of our knowledge, there is no such work on CR-WSNs.

We provide the impetus required for further research to solve energy conservation, load balancing, PU protection, and efficient bandwidth utilization in CR-WSNs.

## Figures and Tables

**Table 1:** Clustering differences between WSNs and CR-WSNs.

Factors	WSNs	CR-WSNs
Channels	Usually single channel (multiple channels possible)	Multiple channels are required
Protection of PU access priority	Not an issue	Must be protected; incumbent license holder for the channels must get priority
Hardware	Readily available (off-the-shelf)	Not readily available; still at the R&D level
Sensor form factor	Small	Moderate to large
Memory	Limited	High
CH	Any node can take a CH role	Mostly dedicated; role changes if all WS nodes have CR
CH as a single point of failure	Relatively less critical	Critical in case of dedicated CH
Computation capability	Moderate	High
Power	Power constraints	Comparatively higher power required
Global addressing scheme	Not required; not recommended	Generally not required, but recommended
Topology change handling	Relatively easy	Difficult
Trust and security	Relatively less critical	Highly critical
Duty cycle	Low	High; required to monitor PU arrival
Bandwidth deficiency	Yes	Generally not an issue
Metrics	Efficiency, resolution, latency, scalability, robustness	PU arrival prediction, channel switching, latency, robustness

**Table 2:** Comparison of the existing clustering schemes for CR-WSNs.

Clustering Scheme	Objectives	Metrics	Performance Enhancement	CH Selection	Special Node
**KoN-MAC**	MAC protocol	Similar to WSNs	Added channel weight	Elected	Gateway node
**MQ-MAC**	MAC protocol	Similar to LEACH	Modified for CRNs	Predetermined probability (similar to LEACH)	Gateway node
**CBSS**	Energy efficiency Cooperative task	Similarity between sensor nodes	Energy consumption	No CH, but selects a node for spectrum sensing	NA
**ECS**	Cooperative spectrum sensing	Spectrum-aware node-grouping	Energy consumption (compared with DEEC algorithm [56] for WSNs	Predetermined probability (similar to LEACH) Only nodes in awake mode engage in the cluster-head selection process	NA
**SCEEM**	Cross-layer routing protocol	Higher spectrum rank	Optimal number of clusters	Based on highest spectrum energy rank	NA
**CENTRE**	Energy efficiency	Distance to the CH	Clustering overhead (compared with DSAC)	Self-declared	Temporary support (TS) node
**EESA-RLC**	Energy efficient PU detection	Optimal number of clusters	Cooperative probability of detection	Number of vacant channels (similar to HEED [57])	NA
**BECHR**	Spectrum sensing	NA	Energy consumption (compared with LEACH)	Iterative (based on energy level)	No, but all nodes have GPS
**CogLEACH**	Clustering evaluation in three different similarity models	Number of vacant channels	Extension of LEACH	Number of vacant channels as a weight in the probability of each node becoming a CH	NA
**DSAC**	Energy efficiency	Optimal number of clusters	Energy consumption Scalability	Iterative (each node has equal probability to become CH)	Only certain nodes can be CHs
**EAC**	Energy efficiency	Optimal number of clusters	NA	Residual energy and number of neighbor nodes	NA
**ESAC/mESAC**	Event-driven clustering	Spectrum availabilities	Energy consumption (with a cost of delay)	Node degree, available channels, and distance to the sink	NA
**LEAUCH**	Energy efficiency	Number of channels available Uneven-sized clustering	Energy consumption Network load balance	Number of idle channels Distance from the sink	NA
**Virtual clustering**	Control channel establishment MAC protocol	Similarities of spectrum opportunities Geographic location	Collision mitigation Control packet success rate Delay	NA	NA
**SCR**	Routing	Spectrum measurements Residual energy	Mean packet delay Energy consumption	Spectrum energy rank	NA
**CR-HDC**	Energy efficiency	Geographic location Optimal transmission range	NA	Iterative (based on residual energy and average one-hop distance to all other nodes)	NA

**NA:** Not applicable or information not available.
